# Short Tools to Assess Young Children's Dietary Intake: A Systematic Review Focusing on Application to Dietary Index Research

**DOI:** 10.1155/2013/709626

**Published:** 2013-09-26

**Authors:** Lucinda K. Bell, Rebecca K. Golley, Anthea M. Magarey

**Affiliations:** ^1^Department of Nutrition and Dietetics, Flinders University, Bedford Park, SA, 5042, Australia; ^2^Department of Nutrition and Dietetics, Flinders Clinical and Molecular Medicine, School of Medicine, Flinders Medical Centre, Flinders University, Adelaide, SA, Australia; ^3^Public Health Group, Sansom Institute of Health Research, University of South Australia, 5000, Australia

## Abstract

Dietary indices evaluate diet quality, usually based on current dietary guidelines. Indices can therefore contribute to our understanding of early-life obesity-risk dietary behaviours. Yet indices are commonly applied to dietary data collected by onerous methods (e.g., recalls or records). Short dietary assessment instruments are an attractive alternative to collect data from which to derive an index score. A systematic review of studies published before April 2013 was conducted to identify short (≤50 items) tools that measure whole-of-diet intake of young children (birth-five years) and are applicable to dietary indices, in particular screening obesogenic dietary behaviours. The search identified 3686 papers of which 16, reporting on 15 tools (*n* = 7, infants and toddlers birth-24 months; *n* = 8, preschoolers 2–5 years), met the inclusion criteria. Most tools were food frequency questionnaires (*n* = 14), with one innovative dietary questionnaire identified. Seven were tested for validity or reliability, and one was tested for both. Six tools (*n* = 2, infants and toddlers; *n* = 4, preschoolers) are applicable for use with current dietary indices, five of which screen obesogenic dietary behaviours. Given the limited number of brief, valid and reliable dietary assessment tools for young children to which an index can be applied, future short tool development is warranted, particularly for screening obesogenic dietary behaviours.

## 1. Introduction

Individuals do not consume single nutrients, foods, or food groups, but rather combinations of foods [1]. Therefore in nutrition research it is appealing to capture the mix of foods and/or nutrients likely to influence health [2]. Dietary indices, for example evaluate diet quality by assessing dietary intake against predetermined criteria, usually reflecting current dietary guidelines [3]. 

Childhood overweight and obesity is a global health problem with 40 million children under the age of five classified as overweight [4]. Given the consequences of obesity and the persistence of obesity from childhood into adulthood [5], it is of major importance to address overweight early in life. As recommendations for overweight prevention and treatment are consistent with food-based dietary guidelines [6, 7], dietary indices offer a way of understanding the contribution of early life food intake to obesity risk.

Evaluation of diet against food-based dietary guidelines using an index [8] still requires accurate assessment of dietary intake at the food or food group level. In children under five, indices have commonly been applied to dietary data collected by 24-hour recalls, diet diaries, or weighed food records [9]. Yet, these methods are associated with high respondent burden and are cost- and time-intensive in terms of administration and analysis [10]. The use of these dietary assessment methods is a challenge in large epidemiological studies. Additionally, while energy and nutrient intakes can easily be derived from these detailed methods, it is often difficult to extract food intake data in a way that allows meaningful comparison with food-based dietary guidelines [8].

Short, simple dietary assessment instruments are an attractive alternative to collect data from which to derive a diet quality score, as they are associated with reduced participant burden, data handling and processing, and costs. They are consequently suitable for survey or epidemiological research [11]. Further, as they supply information quickly [11], they are useful in clinical settings for the rapid assessment of individuals' food intake against food-based dietary guidelines. In view of the high worldwide childhood obesity rates, simple tools that assess early life obesogenic dietary habits are crucial. Given their advantages, short tools that enable evaluation of young children's dietary intake against food-based dietary guidelines using a dietary index are required.

Thus, this review aimed to (1) examine short tools, including their reliability and validity, that measure diet of children birth-five years, (2) identify the short tools that could be used in dietary index research, including screening of obesogenic dietary behaviours.

## 2. Methods

### 2.1. Search and Selection Strategy

**Figure 1:**
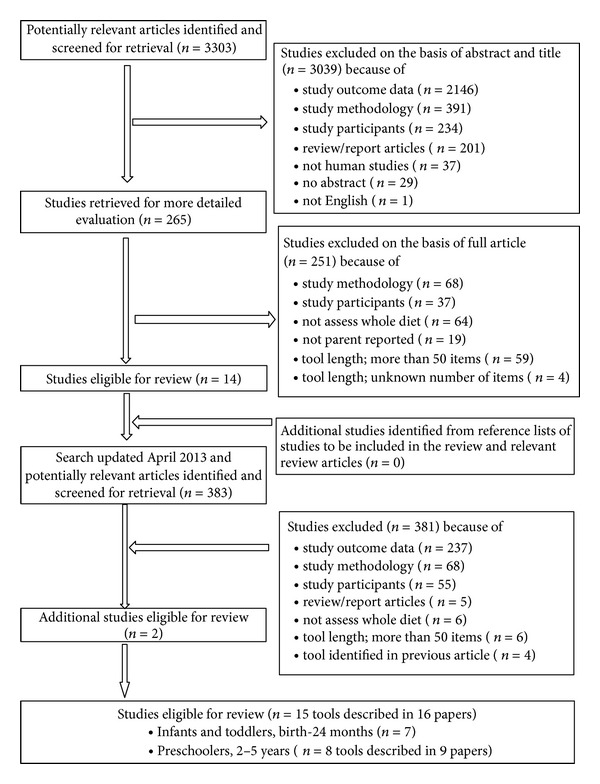
Quorom statement flow diagram. Studies assessing whole-of-diet intake of infants and toddlers and pre-schoolers using a short assessment tool.

A six-stage systematic search was conducted to identify existing short tools that measure whole diet in young children. The search strategy and article selection are summarised in Figure 1. In stage one, MEDLINE via PubMed, Web of Science, and SCOPUS were searched for relevant articles published prior to June 2011. The search terms were developed and combined under the following headings: (1) *child (birth-5 years)*, for example infant, toddler, preschooler, and child; (2) *diet*, for example food, nutrition, dietary intake, dietary pattern, eating pattern, food intake; (3) *assessment tool*, for example tool, dietary assessment, evaluate, questionnaire, checklist, validity, and reproducibility. Search term lists were comprehensive with small adaptations made for individual databases searched (see Supplementary information). Stage two involved elimination of irrelevant articles in Endnote using specific term searches through “title” and “keywords” (all terms presented in the Supplementary Information in Supplementary Material available online at http://dx.doi.org/10.1155/2013/709626). Subsequently, the title and abstract of the remaining 3303 articles were screened against the review inclusion and exclusion criteria, outlined below (stage three). If it was unclear whether an article should be included from the title and abstract, the full article was retrieved and screened (stage four). In stage five, reference lists of all included articles and relevant reviews were searched for additional studies. Lastly, searches were rerun in April 2013 to identify articles published after June 2011 (stage six). All resulting articles were screened according to stages two to five. Overall, all articles were assessed for eligibility independently by the primary author but in consultation with all coauthors.

### 2.2. Inclusion and Exclusion Criteria

The included studies were determined using the following criteria.

1. Types of outcome measures: studies with whole-of diet intake data were included. Those assessing individual foods, food groups, nutrients or behaviours, and/or household, family, or group consumption were excluded.
2. Types of dietary assessment methods: studies assessing dietary intake using a short dietary assessment tool were included. For example food frequency questionnaires, checklists, and other dietary questionnaires classified as 50 food intake questions or less. This criterion was set by the authors in an attempt to capture tools that were five pages or less and/or could be completed within 30 minutes. Articles were excluded if dietary assessment tools such as 24-hour recalls, diet histories or food records were used to measure food intake, as they are considered standardised methods that are limited by complex researcher-based administration [12]. If the number of questionnaire items was not reported, or if the tool had been captured in a previously identified paper, articles were excluded.
3. Types of participants: studies assessing dietary intake of healthy children aged birth to five years, reported by a parent or primary caregiver without assistance from the child, were included. Studies not applicable to the general population (e.g., preterm infants or children with disabilities, health conditions, or behavioural/learning difficulties) were excluded.
4. Other: studies were limited to the English language, humans and those with an abstract. Review studies, reports, conference papers, and similar documents were excluded.

### 2.3. Data Extraction and Analysis

Data, including sample characteristics, questionnaire details, and reliability and validity were extracted into standardized tables by the principal author and checked for completion and accuracy by all coauthors. Data synthesis comprised grouping studies by age group and comparing in terms of dietary assessment characteristics; reliability (i.e., tool reproducibility or repeatability using a test-retest procedure [13]); validity (i.e., the ability to accurately measure food consumption data that represents the true intake of the individual [14], determined by comparison with an already validated method); and usefulness for current dietary index applications and screening obesogenic dietary behaviours. Applicability of tools to dietary indices was determined by comparing tool characteristics with characteristics of available indices for children aged up to five years, based on those identified in a recent review [9]. Tools were defined as applicable to dietary indices if all index components could be assessed both easily and accurately. Indices covering the five “core” food groups (i.e., foods recommended to be consumed every day including fruits; vegetables; cereals (e.g., bread, rice, and pasta, noodles); meat and alternatives (e.g., fish, eggs, and nuts); dairy), are highlighted. Indices suitable for screening obesogenic dietary behaviours were defined by the assessment of foods not included in the “core” food groups, described as “noncore” (energy-dense, low nutrient) foods and recommended to be consumed in minimal amounts [6, 15].

## 3. Results

### 3.1. General Description of Included Studies

Sixteen studies met the review inclusion criteria (Figure 1). The most common reason for exclusion was the type of outcome data (*n* = 2383), followed by study assessment methodology (*n* = 526) and study participants (*n* = 322). The final 16 papers reported on 15 tools developed to assess dietary intake in early childhood (birth-5 years); seven evaluate infant and toddler dietary intake [16–22] eight evaluate preschoolers dietary intake [23–31] (Table 1). Studies included a range of population groups from predominately European [16, 17, 19–21, 24–28] and other western countries [18, 23, 29–31] and were largely published from 2006 onwards [16, 18, 19, 23–31], with no retrieved papers published prior to 2000. The number of participants varied from 44 [25] to 27 763 [17], with three studies presenting data from large, prospective birth cohorts UK Southampton Women's Study (SWS) [19], UK Avon Longitudinal Study of Parents and Children (ALSPAC) [16], and the Norwegian Mother and Child Cohort Study (MoBa) [17].

### 3.2. Dietary Assessment Methods and Testing

Most (*n* = 14 of 15) tools used a food frequency questionnaire (FFQs) format [16–28, 30, 31], with one innovative tool, the NutriSTEP nutrition screening tool for preschoolers, identified [29]. The majority of tools were self-administered [16–18, 20, 21, 23–29, 31] and nonquantitative [16–18, 22–25, 29, 31]. The average tool length was 33 items (range 6–47), with 5 tools comprising less than 25 items [18, 20, 21, 23, 29]. Reference periods for recalling foods varied from the past week [18, 19, 22] to past year [27, 28]. Fourteen of the 16 studies reviewed reported food or food group intake as a tool outcome measure [16–18, 20–27, 29–31], whilst two reported energy and nutrient intakes only [19, 28]. Overall, testing was undertaken on approximately half of identified tools (*n* = 7/15, described in 8 papers) (Table 2). A range of tests to assess reliability and validity were reported. Test definitions and review assessment criteria are presented in the Supplementary Table. Validity (Table 3) and/or reliability (Table 4) were most commonly tested using correlations, although agreement statistics were also used. 

#### 3.2.1. Infants and Toddlers (Birth-24 Months)

All seven [16–22] tools assessing infant and toddler dietary intakes were FFQs, ranging in length from 15 [20] to 43 [16] items. Three tools were evaluated for validity [19–21] (Table 4) whilst none were evaluated for reliability.

Validity testing revealed that the FFQs overestimated energy and nutrient intakes compared with the selected reference standard (all weighed dietary records, WDR) [19–21]. Correlations for energy and nutrients were low to moderate and slightly higher when energy adjusted [20, 21]. Bland Altman plots for nutrient intakes showed mostly positive mean differences [19], systematic increases in difference with increasing intake for most nutrients [20, 21] and large limits of agreement [20, 21]. Little gross misclassification (3% [21], 5% [20]), defined as classification of intake by the tool in the opposite quartile or tertile of intake, was reported with over one-third of subjects (38% [21], 36% [20]) classified into the same category of nutrient intake. At the food level, FFQ's generally revealed higher median intakes for several food items (11/17 [21] and 7/15 foods [20]) than the WDR [20, 21]. Correlations for most foods were low or moderate with low (*r* = 0.48 [20]) and moderate (*r* = 0.62 [21]) overall median correlations. Importantly, no studies used agreement statistics at the food level.

#### 3.2.2. Preschool Children (2–5 Years)

Of the eight tools evaluating pre-schoolers' dietary intakes, described in nine papers [23–31], seven were FFQ's [23–28, 30, 31] but length varied widely (six [29] to 47 [27, 28, 31] items). Overall, three tools were assessed for reliability only [24, 25, 29] and one for reliability and validity of food [27] and nutrient [28] intake (Tables 3 and 4).

To assess test-retest reliability [24, 25, 27–29] the period between administrations varied, ranging from two to four weeks [29] to an average of four months (range 0–364 days) [24]. No tool was assessed for reliability of energy intake and only one for nutrients [28]. The latter revealed that for average daily calcium intakes readministrations were not significantly different (*P* = 0.26), were highly correlated (*r* = 0.80) with moderate agreement (*k* = 0.60) and that nearly all subjects were classified into the same or adjacent quartile of intake (93%) [28]. The reproducibility of food intake was assessed for four tools [24, 25, 27, 29] and showed no statistically significant differences for most foods (38/43 [24], 13/13 foods [27]). Mean spearman's correlations were moderate (*r* = 0.59 [24], *r* = 0.62 [25], and *r* = 0.64 [27]) with good intraclass correlation coefficients (ICC's) reported for many food items (*n* = 28/39 [25]; *n* = 13/13 [27]) and moderate overall mean ICC's (*r* = 0.59 [25, 27]). Two studies showed moderate overall agreement for food items (*k* = 0.48 [24], *k* = 0.55 [29]).

Only one tool was assessed for validity, reported in two studies [27, 28]. This tool significantly underestimated calcium intake measured by an estimated dietary record (EDR), yet methods were moderately correlated (*r* = 0.52, adjusted *r* = 0.59) [28]. Sensitivity and specificity of calcium intake was 62% and 77%, respectively, [28] and nearly half (42%) of subjects were correctly classified [28]. Agreement statistics showed fair agreement (*k* = 0.38) and large differences for higher average nutrient intakes (Bland-Altman plot) [28]. For food intake, mean differences were predominately less than 30% (12/13 foods) [27], whilst the median correlation was low (*r* = 0.48 [27]) and agreement mostly poor (4/13 foods) or fair (4/13 foods) [27]. Gross misclassification was less than 10% for all food groups whilst classification into the same or adjacent category ranged from 67% (meat products) to 88% (fruit juice) [27].

### 3.3. Dietary Index Applications

Dietary indices developed to characterise the diet quality of infants, toddlers or preschool-aged children are summarised in Table 5 [26, 31–48]. Overall, data from six tools (*n* = 2, infants and toddlers [20, 21]; *n* = 4, pre-schooler [26, 27, 30, 31]) can be applied to five measures of diet quality reviewed [26, 31, 39, 42, 47], all developed for use in pre-schoolers (Table 5). Two have been tested for validity only [20, 21] and one for both validity and reliability [27]. Of these six short tools, two [26, 31], both for use in pre-schoolers, have previously been used in dietary index applications. The Healthy Nutrition Score for Kids and Youth (HuSKY) has been applied to the 54-item (45 food-item) semi-quantitative FFQ assessing intakes of three to six-year-old German children [26], whilst the 47-item non-quantitative FFQ has been used to assess dietary diversity in American children under five [31].

No other short tools were identified that provide dietary data to which a dietary index could be applied, often because the level of detail provided by the tool was too minimal for application of an index. This is particularly evident for those indices comprising food-group subcategories (e.g., “vitamin A-rich vegetables”) [33, 36, 38, 43, 44, 48]. Additionally, application of several tools to current indices would require detailed analysis to determine nutrient (e.g., total fat, cholesterol, and iron) intakes [32, 34, 40, 41, 43, 44, 46]. Lastly, portion size quantification is required for the majority of dietary indices reviewed [26, 32, 34, 36, 38–44, 46–48] and thus only quantitative or semiquantitative tools provide data to which these indices could be applied. 

### 3.4. Screening Obesogenic Behaviours

Of the 15 tools reviewed, 13 assess the intake of “noncore” foods and/or beverages (*n* = 6, infant and toddlers [16–21]; *n* = 7 pre-schoolers [23–30]). Three of these were specifically designed to screen obesity related behaviours [24, 25, 29] whilst five were identified (above) as being useful for application of a dietary index. Of the 19 indices reviewed [9], three (*n* = 1, infants and toddlers [34]; *n* = 4, pre-schoolers [26, 39]) included food items associated with poor diet quality, such as intake of high fat or sugary foods and/or beverages. Two of these indices can be used with the short tools identified in this review [26, 39].

## 4. Discussion

This review identified 16 papers reporting on 15 short dietary assessment tools that measure whole diet of children under five years (*n* = 7, infants and toddlers; *n* = 8, pre-schoolers). Tool reliability and validity and applicability to dietary indices and for screening obesogenic dietary behaviours are highlighted. All but one tool was a FFQ, and approximately half (*n* = 7) of all tools were tested for either reliability or validity, and one tested for both. Six tools provide dietary intake data to which an index can be applied, five of which screen obesogenic dietary behaviours. Overall, testing of tool properties was limited and few tools are applicable to current dietary indices that screen obesogenic dietary behaviours of children from birth to five years of age.

Of the 15 tools identified in this review, only seven were tested for validity and/or reliability at the food or food group level. In general, there was a lack of reliability testing to accompany validity testing with only one of four tools assessed for validity also assessed for reliability. As validity requires reliability [49], the remaining three tools cannot be identified as valid. Moreover, there was a high reliance on correlations which assess association only and thus should not be used alone but alongside agreement measures such as kappa statistic and Bland-Altman analysis [50, 51]. Further, although the reference period covered by the validation standard should correspond to that of the questionnaire [52], 3- or 7-day food records were commonly used in the reviewed studies to assess the validity of FFQs covering two weeks [20, 21] or 12 months intake [27, 28]. For reliability studies, if readministration periods are too close, subjects may remember their previous responses, or if too far apart, lower reliability may reflect true variation in diet [52], particularly in young children at an age when dietary habits are rapidly changing [53]. This is evident as an average re-administration period of 4 months yielded weaker agreement [24] than studies with shorter re-administration periods. Despite these limitations in tool testing, and in considering the realistic estimates of measurement error between two dietary assessment methods [54] in conjunction with unstable dietary habits of young children, the reliability and validity results presented here can be considered reasonable. Thus, several short dietary assessment tools can be judged as useful for characterising the diet of children under 5.

Given the increasing interest in assessing diet quality using an index, resulting from an increased understanding of the complexity in which individuals consume foods [55], determining those short tools that are useful for dietary index applications is of interest. For the current indices available for children under five years of age, summarised in this review, diet quality is assessed based on intake of particular foods or food groups, nutrients, or a combination of both. Although most of the tools reviewed estimate whole-of-diet food intake making them potentially useful for food or food-group based index applications, few (*n* = 6 of 15) can be directly applied to current indices of diet quality [20, 21, 26, 27, 30, 31]. Further, these tools are limited by a lack of testing, with only one tested for reliability and validity [27, 28]. Thus the accuracy of the other five tools in assessing dietary intake, and diet quality when applied to an index, is questionable. Therefore, testing of tool properties is recommended prior to dietary index applications.

Several factors explain why other short tools reviewed are not useful for dietary index applications. First, as mentioned, many indices assess diet quality based on nutrient intakes or a combination of nutrient and food intakes. Applying an index of this type to a questionnaire-type tool requires linkage with appropriate food composition data to derive nutrient intakes. Alternatively, questionnaire-type tools are most applicable to food-based indices. Further, several indices assess food-group subcategories, such as “vitamin A-rich vegetables” or “dark green vegetables,” which are not measured by the short tools reviewed. Also limiting applicability is that portion size quantification is required to apply dietary data to several indices. Although these factors limit the applicability of short tools to current indices, several tools that capture food groups of interest are ideal for development of a suitable index. For example the 47-item FFQ by Huybrechts et al. [27] is suitable as it assesses “core” and “noncore” food intake and was the one tool tested for both reliability and validity of food intake. Development of a dietary index based on food intake assessed using this short tool would be appropriate. Alternatively, future research to develop suitable short dietary assessment tools that measure whole diet to which a current index can be applied is ideal.

Moreover, in view of the high rates of overweight and obesity among children under five worldwide [4], indices are potentially a useful tool to evaluate early life dietary behaviours that contribute to obesity risk. Yet few current indices for children less than five years assess obesogenic dietary behaviours, with many evaluating “core” food and/or nutrient intakes only. Thus, future indices based on “core” and “noncore” food intake are warranted. Additionally, considering that few short tools assess “noncore” intakes and are useful for application of a dietary intake there is a need for future development of short tools that are useful for both dietary index applications and screening obesogenic dietary behaviours in children under five, particularly in those less than two years of age.

Overall, this systematic review highlights the lack of high quality short dietary intake assessment tools for young children, particularly less than two years, to which a dietary index can be applied. Further, as the majority of those tools available for dietary index applications were developed and tested in European populations, restricting their generalisability outside the European context, there is a need for short dietary assessment tools developed for use in other populations of young children to which an index can be applied. Lastly, it is important to note that several rapid dietary assessment tools have been designed for use in young children, yet are not presented in this review as they focus on limited aspects of food intake, for example fruit and vegetables [56], beverages [57], and obesity-related food and beverages only [58], not total diet. Future rapid dietary assessment tools should be designed to comprehensively measure young children's whole-of-diet intake, including obesogenic dietary behaviours, and should be tested for reliability and validity of food intake.

## 5. Conclusion

A key finding of this review is that although several short dietary assessment tools were identified as useful for characterising whole diet of children birth-5 years, there is an overall lack of brief, valid and reliable dietary assessment tools available for use in this age group. This highlights a need for greater testing of existing short tools. A second key finding is that few short dietary assessment tools, particularly those developed for under 2's, are suitable for dietary index applications and for screening obesogenic dietary behaviours of young children. Due to the benefits of assessing diet quality using indices and of capturing dietary intake using less demanding, time-consuming and expensive dietary assessment methodologies, this review identifies opportunities for short tool development for use in children under five that are adequately reliable and valid for use, applicable to dietary indices, and that assess obesogenic dietary behaviours.

## Supplementary Material

Supplementary 1 material provides details of the six-stage search process undertaken for the literature review. Details of inclusion and exclusion criteria applied to retrieved articles are detailed in the table. Supplementary 2 defines statistical tests used to assess reliability and validity of reviewed studies and highlights the review assessment criteria.

## Figures and Tables

**Table 1:** Characteristics of included studies (*n* = 16) and their tools (*n* = 15).

Reference, details, country	Age diet assessed, sample size (gender)	Dietary intake measurement						Outcomes (food, energy, and/or nutrient intakes)
		Type and name (if provided) of tool^a^	Number of food items	Tool reference period	Self- or interviewer administered^b^	Number of response categories (range)	Other tool details	
Infants and toddlers (birth-24 months)								
Smithers et al. (2012) [16]; UK	6 mo, *n* = 7052 (NR)	Nonquantitative FFQ	43	“nowadays”	Self	Report “*x*” times a week	Items include milk drinks (including formula, BM), cereals (baby, other), rusks, bread/toast, biscuits, ready-to-eat meat/fish/vegetables/baby puddings (fruit, milk), home-cooked meat/fish/vegetables/potatoes/other vegetables/puddings (fruit, milk), raw fruit/vegetables, beverages (juice, fizzy drinks, tea, coffee, and water), sweets, crisps, and chocolate	Foods

Ystrom et al. (2009) [17]; Norway	18 mo, *n* = 27763 (51% boys)	Nonquantitative FFQ	36	“Current diet”; NFS	Self	Drinks, (never to ≥5 times/day); Foods, (never to ≥3 times/day)	Items include dairy products (milk, yoghurt), meat, fish, fruit, vegetables, potato, porridge, bread, rice, water, fruit juice, soda, chocolate, sweets, desserts, and cakes	Foods

Dee et al. (2008) [18]; USA	6 mo, *n* = 1984 (NR)	Nonquantitative FFQ	21	1 wk	Self	Report number of feedings per day or per week	Items include milk (BM, formula, cows, rice, goat, and soy), other dairy (yoghurt, cheese, ice-cream, and pudding), other soy foods (tofu, soy desserts), fruit and vegetable juice, sweet drinks, baby cereal, other cereals (breakfast cereals, biscuits, breads, rice, pasta, etc.), fruit, vegetables, French fries, meat and chicken, fish, nut-based foods, eggs, sweet foods (candy, cookies, cake, etc.), and other	Foods nutrients

Marriott et al. (2008) [19]; UK	6 mo, *n* = 50 (50% boys)	Quantitative FFQ	34	1 wk	Interviewer	Open responses	Items include meat, fish, vegetables, fruits, cereals, snack foods, commercial baby foods, non-milk drinks and human milk, baby formulas, and other milks. Portion size estimated using household measures	Energy nutrients

Andersen et al. (2004) [20]; Norway	24 mo, *n* = 187 (53% boys)	Semi-quantitative FFQ	15	2 wk	Self	Not specified (never/<1/month to several times/day)	125 foods grouped into 15 questions based on the Norwegian meal pattern. Items include dairy (milk, yoghurt, and cheese), bread, potatoes, vegetables, fruit, meat, fish, cake, chocolate, and soft drinks. Other questions on dietary supplements, food habits, and child nutrition information sources. Portion size estimated using a photographic booklet with four different sized (small–large) or household units (e.g., slices, pieces, and spoons)	Foods Energy Nutrients

Andersen et al. (2003) [21]; Norway	12 mo, *n* = 64 (58% boys)	Semiquantitative FFQ	18	2 wk	Self	Not specified (never/<1/month to several times/day)	140 foods grouped into 18 questions based on the Norwegian meal pattern. Items include dairy (milk, yoghurt, and cheese), baby cereal, bread, potatoes, vegetables, fruit, meat, sweetened drinks; and commercial baby foods. Other questions on dietary supplements food habits, child nutrition information sources. Portion size estimated using a photographic booklet with four different sized (small–large) or household units (e.g., slices, pieces, and spoons)	Foods Energy Nutrients

Lartey et al. (2000) [22]; Ghana	1–6 mo, *n* = 216 (53% girls)	Nonquantitative FFQ	28	1 wk	NR	NR	Items include porridges, fruits, vegetables, soups, cereals, legumes, roots and tubers, animal products, cereal-legume mixtures, and cereal-animal product mixtures. Other questions on breastfeeding frequency and daily number other milk feedings	Foods

Preschoolers (2–5 years; 25–60 months)								
Pabayo et al. (2012) [23]; Canada	4-5 y, *n* = 2015 (51.5% boys)	Nonquantitative FFQ	20	Usual intake; NFS	Self	Report total number of daily or weekly servings	Items include fruits, vegetables, grain products (bread, cereal, pasta, and rice), milk and alternatives (white or flavoured, soy or rice beverages, cheese, and yogurt), and meat and alternatives (meat, poultry, fish, peanut butter, nuts, and tofu), chips, French fries, candy, chocolate, regular soft drinks, and cakes and cookies	Foods

Lanfer et al. (2011) [24]; IDEFICS consortium; European countriesc	2–9 y (2–<6y, 39.5%; 6–<10 y, 60.5%), *n* = 258 (44% boys)	Nonquantitative FFQ; Children's Eating Habits Questionnaire (CEHQ-FFQ)	43	4 wk	Self	8 (never/<1/week to ≥4/day and “I have no idea”)	Items include vegetables, potatoes, fruit, meat, fish, egg, cereals, bread, pasta, dairy (cheese, milk, and yoghurt), sweetened beverages, spreads, sauces, take-away products, salty snacks, chocolate, candy, cake, and ice-cream. Screening instrument investigating food consumption frequency and behaviours associated with child overweight, obesity, and general health	Foods

Ebenegger et al. (2010) [25]; Switzerland	Mean 5 y, *n* = 44 (64% boys)	Nonquantitative FFQ	39	4 wk	Self	7 (NR)	Items include fruit, vegetables, potato, meat, fish, dairy (yoghurt, cheese, and dairy desserts), bread, cereal, sauces, sweets and snacks (e.g., chocolate), and drinks (e.g., cola). Other questions on eating habits	Foods

Kleiser et al. (2009) [26]; Germany	3–17y, (3–6 y, 7–10 y, 11–17 y), *n* = 14105 (51% boys)	Semiquantitative FFQ	45	Previous “few wks”; NFS	Self	10 (never to >5/day)	Items include vegetables, fruit, fish, bread/cereal, rice/pasta/potatoes, milk/dairy products, eggs, meat, fats, sweets/fatty snacks/soft drinks, and other beverages. Other questions on eating habits, supplement intake, fortified foods, light products, convenience food, and probiotic products. Portion size estimated using illustrations or standard household measures	Foods energy nutrients

Huybrechts et al. (2009) [27]; Belgium Huybrechts et al. (2006) [28]; Belgium	2.5–6.5 y, *n* = 650 validity *n* = 124 reproducibility (NR) 2.5–6.5 y, mean 4.5 y *n* = 1052, (50% boys)	Semiquantitative FFQ	47	12 mo	Self	6 (every day to never or less than 1 day/month)	Items include beverages (water, juice, and milk drinks), dairy (cheese, yoghurt), meat and meat alternatives (fish, eggs), bread, pasta, rice, vegetables, fruit, potatoes (including fried), meat/fish products, chocolate, sweet snacks, salty snacks, and desserts. Other questions on food habits of some product groups. Portion size estimated using examples of common standard measures	Foods Energy Nutrients

Randall Simpson et al. (2008) [29]; Canada	3-4 y, *n* = 269 validity *n* = 140 reproducibility (94% girls)	Nonquantitative screening tool; NutriSTEP	6	Usual intake; NFS	Self	NR	Items include grains, milk, fruit, vegetables, meat, and fast food. Other questions on nutrition risk constructs: physical growth, physical activity and sedentary behaviour, and factors affecting food intake	Foods

Romaguera et al. (2008) [30]; Argentina	2–9 y (mean boys = 5.1; girls = 5.2), *n* = 360 (NR)	Semiquantitative FFQ	46	NR	Interviewer	NR	Items include cereals/grains, potatoes/tubers, pulses, fish, meat/meat products, eggs, milk/dairy products, fruits and vegetables, fats, added oil, sugary drinks, herbal teas, and added sugar and sweets, sweet and milky desserts. Portion sizes determined according to the observed amount usually consumed in population, measured prior to study.	Foods energy nutrients

Sullivan et al. (2006) [31]; USA	<60 mo, *n* = 191 (59% boys)	Nonquantitative FFQ	47	2 mo	Self	9 (1, 2, and 3/day; 1, 2, 3/week; 0, 1, and 2/month)	Items include fruits, vegetables, legumes and nuts, dairy products, meat, fish, and poultry.	Foods

FFQ: food frequency questionnaire; iDEFICS: Identification and prevention of dietary- and lifestyle-induced health effects in children and infants; NFS: not further specified; mo: months; NR: not reported; USA: United States of America; UK: United Kingdom; y: years.

^a^Tools were defined as quantitative (quantity of food consumed was estimated using weights, measures, or food models), semiquantitative (quantity of food consumed estimated using a standard portion size, serving, or a predetermined amount and respondent asked about the number of portions consumed), or nonquantitative (quantity of food consumed not assessed).

^b^Self-administered (primary caregiver completed the dietary assessment without assistance); interviewer-administered (a trained interviewer elicited the dietary assessment information from the primary care-giver in a one-on-one setting).

^c^Italy, Estonia, Cyprus, Belgium, Sweden, Germany, Hungary, and Spain.

**Table 2:** Summary of availability of validity and reproducibility data for each study according to energy and/or nutrient intake and food intake.

Reference details	Validity		Reliability	
	Energy and/or nutrients	Foods	Energy and/or nutrients	Foods
Infants and toddlers (birth-24 months)				
Smithers et al. (2012) [16]	—	—	—	—
Ystrom et al. (2009) [17]	—	—	—	—
Dee et al. (2008) [18]	—	—	—	—
Marriott et al. (2008) [19]	*√*	—	—	—
Andersen et al. (2004) [20]	*√*	*√*	—	—
Andersen et al. (2003) [21]	*√*	*√*	—	—
Lartey et al. (2000) [22]	—	—	—	—
Preschoolers (2–5 years)				
Pabayo et al. (2012) [23]	—	—	—	—
Lanfer et al. (2011) [24]	—	—	—	*√*
Ebenegger et al. (2010) [25]	—	—	—	*√*
Kleiser et al. (2009) [26]	—	—	—	—
Huybrechts et al. (2009) [27]	—	*√*	—	*√*
Huybrechts et al. (2006) [28]	*√*	—	*√*	—
Randall Simpson et al. (2008) [29]	—	—	—	*√*
Romaguera et al. (2008) [30]	—	—	—	—
Sullivan et al. (2006) [31]	—	—	—	—

**Table 3:** Short dietary assessment tool validity studies among infants and toddlers (birth-24 months) and preschoolers (2–5 years).

Reference details; tool length; validation standard; reference period; sample size					
	Infants and toddlers (birth-24 months)			Pre-schoolers (2–5 years)	
	Marriott et al. (2008) [19]; 34 items; 4 d WDR; 15 days; *n* = 50	Andersen et al. (2003) [21]; 18 items; 7 d WDR; 1-2 weeks; *n* = 64	Andersen et al. (2004) [20]; 7 d WDR; 15 items; 1-2 weeks; *n* = 187	Huybrechts et al. (2009) [27]; 47 items; 3 d EDR; 1 week; *n* = 650	Huybrechts et al. (2006) [28]; 47 items; 3 d EDR; 1 week; *n* = 1052
Energy and nutrients					
Mean/median nutrient intakes	All median intakes significant higher (*P* < 0.05), except sodium	All median intakes significant higher (*P* < 0.05), except Ca	All median intakes significant higher (*P* < 0.05), except protein, Carb, SFA, and Ca	—	Significantly lower mean Ca intake: 777 mg/d v 838 ± 305 mg/d; difference 61 ± 294 mg/d (*P* < 0.001)

Mean/median nutrient densities	—	No significant differences except for protein, SFA, MUFA, fibre, vitamin A, vitamin C, calcium, and iron	No significant differences except for protein, SFA, MUFA, fibre, vitamin A, vitamin C, calcium, and iron	—	—

Pearson's correlation	—	—	—	—	*r* = 0.52, corrected for intravariability: *r* = 0.59

Spearman's correlation (nutrients)	*r* = 0.63 (range 0.39–0.86) *energy-adjusted*: *r* = 0.55–0.89	*r* = 0.50 (range 0.18–0.72) *energy-adjusted*: *r* = 0.50 (0.16–0.79)	*r* = 0.38 (range 0.26–0.50) *energy-adjusted* *r* = 0.52 (range 0.46–0.66)	—	—

Spearman's correlation (foods)	—	*r* = 0.62 (range 0.28–0.83)	*r* = 0.48 (range 0.26–0.69)	*r* = 0.48 (range 0.23–0.62) *corrected*: *r* = 0.32–0.75	—

Specificity	—	—	—	—	77%

Sensitivity	—	—	—	—	62%

Bland Altman, mean bias	Mostly positive, all nutrients within range −12.5% to 12.5%, except vitamin B12 (−18.9%).	Systematic increase in difference with increasing intake, except Ca	Systematic increase in difference with increasing intake for most nutrients	—	Large differences, higher for greater mean intakes

Bland Altman, limits of agreement	—	Large for all nutrients	Large for all nutrients	—	—

Cross classification; nutrients	—	Same quartile, 38% (range 22% fibre—56% SFA); opposite, 3%	Same quartile, 36% (range 29% fat—44% vitamin A); opposite, 5% Energy-adjusted: same, 42%; opposite, 4%	—	Same quartile, 42%; within one, 83%; opposite, 2.4%; difference between quartiles *P* < 0.001

Foods					
Mean/median food group intakes	—	—	—	Mean differences within ±10% 6/13 food groups, 11–30% 6/13, >40% 1/13; median differences within ±10% 5/13 food groups, 11–20% 1/13, >20% 6/13; 100% for 1/13	—

Wilcoxon signed rank test	—	Significantly higher intakes 11/17 food groups, NS differences 6/17	Significantly higher intakes 7/15 food groups, significantly lower intakes 3/1, NS difference for 5/15	Significantly different intake distribution for 6/13 (*P* < 0.01) or 9/13 (*P* < 0.05) food groups; higher 5/13, lower 4/13, NS difference 4/13	—

Kappa statistic	—	—	—	<0.20 4/13 food groups, 0.20–0.40 4/13, 0.41–0.60 2/13, NR 3/13	0.38 (95% CI 0.34, 0.42)

Bland Altman, mean bias	—	—	—	Increasing bias with increasing intakes for “many foods” (*n* not reported)	—

Cross classification; foods	—	—	—	Same = NR, within one = 67%–88%, opposite <10% (2% fruit, fruit juices, and milk products—9% meat products)	—

Ca: calcium; carb: carbohydrates; d: day; EDR: estimated dietary record; LOA: limits of agreement; MUFA: monounsaturated fatty acids; NR: not reported; NS: not significant; %: percent; PUFA: polyunsaturated fatty acids; SFA: saturated fatty acids; WDR: weighed dietary record.

**Table 4:** Short dietary assessment tool reliability studies among preschoolers (2–5 years).

Reference details; tool length; readministration period; sample size					
Tests	Lanfer et al. (2011) [24]; 43 items; 0–354 days, average 4 months; *n* = 258	Ebenegger et al. (2010) [25]; 39 items; within 4-weeks; *n* = 44	Huybrechts et al. (2009) [27]; 47 items; 5 weeks; *n* = 124	Huybrechts et al. (2006) [28]; 47 items; 5 weeks; *n* = 124	Randall Simpson et al. (2008) [29]; 5 items; 2–4 weeks; *n* = 140
Mean/median differences (foods)	—	—	Mean intakes 12/13 food groups within ±10%, 1/13 >10% (11%). Intakes generally lower first administration. Median intakes 10/13 within ±10%, 3/13 > 20%	—	—

Paired *t*-test	—	—	—	*P* = 0.26; 23.8 ± 161.2 mg ca/d (95% CI 17.8, 65.5; 774 ± 252 v 751 ± 255)	*P* < 0.001

Pearson's correlation	—	—	—	*r* = 0.80 for Ca	—

Spearman's correlation (foods)	*r* = 0.59 (range 0.32–0.76); *P* < 0.001 (*r* < 0.50 for 8/43 foods, 0.51–0.69 for 26/43, *r* > 0.70 for 9/43); readministration >4 months (0.28–0.73), <4 months (0.31–0.87)	*r* = 0.62 (*r* < 0.50 for 8/39 (7 *P* < 0.05 and 1 NS), 0.50-0.70 for 22/39 (all *P* < 0.01), >0.70 for 9/39 (all *P* < 0.01)	*r* = 0.64 (*r* = 0.5–0.7 for 10/13, >0.7 for 3/13)	—	—

ICC	—	0.59 (>0.50 28/39 foods)	0.59 (>0.50 13/13 foods)	—	—

Kappa statistic	0.48 (0.23–0.68)	—	—	0.60 (95% CI 0.49–0.71)	0.54 (0.39–0.71)

Wilcoxon signed-rank test:	*P* < 0.05 for 5/43 items, NS for 38/43 items	—	NS for 13/13 foods		

Cross classification	—	—	—	Grossly misclassified = 0%, correctly classified = 56.7%, and adjacent quartile = 36.7%	—

Abbreviations: Ca, calcium, NS, not significant.

**Table 5:** Studies examining diet quality indices among infants and toddlers (birth-24 months) and preschoolers (2–5 years), details of the content of the indices and their applicability to short dietary assessment tools identified in Table 1.

Index name; reference details; age of sample	Index properties			Applicability to short tools identified in Table 1			Can be applied to dietary data assessed by short tools reviewed (Table 1)
	Number of components: component labels	Assesses		Requires		
		Five “core” food groups	“Noncore” foods	Assessment of food-group subcategories	Detailed nutrient analysis	Portion size quantification^a^
Infants and toddlers (birth-24 months)							
Mean Adequacy Ratio (MAR); Hoerr et al. 2006 [32]; 11–25 m	Nutrients included in ratio score vary according to research interests. 8 key nutrients used in [32]	—	—	—	*√*	*√*	N

Dietary Diversity Score, international; Dewey et al. 2006 [33]; 1-2 y	8 or 9 food groups: cereals, roots and tubers, vitamin A-rich fruit and vegetables, other fruit and vegetables, legumes and nuts, meat and alternatives, fats and oils, dairy, and eggs, (fruits and vegetables separate for 9-food group DDS)	*√*	—	*√*	—	—	N

Healthy Eating Index-Canada (HEI-C); Glanville and McIntyre 2006 [34]; 1–3 y	9: grains, fruit and vegetables, milk, meat, other foods (high in fat, sodium, and sat fat), total fat, saturated fat, cholesterol, and variety	*√*	*√*	—	*√*	*√*	N

Food Variety Score (FVS), South Africa; Steyn et al. 2006 [35]; 1–3 y	1: dietary diversity. One point for every food item consumed over 24-hour period from 45-item list^b^.	—	—	—	—	—	N

Diet Quality Score 2 (DQS2), USA; Caliendo et al. 1977 [36]; 1–4 y	6: vegetables, fruit, breads and cereals, meat and milk, citrus fruit, dark green, and yellow vegetables	*√*	—	*√*	—	*√*	N

Child Feeding Index; Ruel et al. 2002 [37]; 1–3 y	7: breastfeeding, does not use bottle^c^, dietary diversity, food frequency, (egg/fish/poultry), food frequency (meat), food, frequency (grains/tubers), and meal frequency	—	—	—	—	—	N

Nutrient Adequacy Score; Krebs-Smith and Clark 1989 [38]; 1–3 y	12: milk and milk products, whole grains, enriched grains, total grains, citrus fruit, other fruit and vegetables, total fruit, green and yellow vegetables, starchy vegetables, other vegetables, total vegetables, and meat and alternatives	*√*	—	*√*	—	*√*	N

Preschoolers (2–5 years)							
Crombie et al. 2009 [39]; 2 y	vegetables; dairy products; meat, fish or alternatives; high-fat or high-sugar snacks	*√*	*√*	—	—	*√*	Y [20, 21, 26–28, 30]

Nutrient Quality Index (NQI), Germany; Libuda et al. 2009 [40]; 2–4 y	17: nutrients: vitamins A, E, K, B6, B12, and C, thiamine, riboflavin, niacin, pantothenic acid, folate; minerals calcium, magnesium, iron, phosphorus, potassium, and zinc	—	—	—	*√*	*√*	N

Healthy Eating Index (HEI), USA; Manios et al. 2009 [41]; 2–5 y	10: Grains, vegetables, fruits, milk, meat, total fat (% calories), saturated fat (% calories), total, cholesterol, sodium, and variety	*√*	—	—	*√*	*√*	N

Servings/day, USA; Kranz et al. 2009 [42]; 2–5 y	5: fruit, vegetables, grains, milk/dairy, meat/alternatives	*√*	—	—	—	*√*	Y [20, 21, 26–28, 30]

HEI-2005, USA; Fungwe et al. 2009 [43]	12: whole fruit (not juice), total vegetables, dark green and orange vegetables and legumes, total and alternatives and beans, food oils, saturated fat, sodium, extra calories from solid fats (including fat in milk), and added sugars	*√*	*√*	*√*	*√*	*√*	N

Healthy Nutrition score (HuSKY); Kleiser et al. 2009 [26]; 3–6 y	11: beverages, vegetables, fruit, fish, breads and dairy products, eggs, meat and sausage, fats and oils (butter/margarine), sweets and fatty snacks, and soft drinks	*√*	*√*	—	—	*√*	Y [26]

Revised Children's Diet USA; Kranz et al. 2008 [44] Kranz et al. 2006 [45]; 2–5 y	13: added sugar, total fat, fat quality—linoleic, fat docosahexaenoic, total grains, whole grains, vegetables, fruits, 100% fruit juice, dairy, iron intake, and energy balance	—	*√*	*√*	*√*	*√*	N

Dietary Diversity Score; Sullivan et al. 2006 [31]; <5y	Dietary diversity. 7: grains-roots-tubers, legumes and nuts, dairy, meat-poultry-fish-eggs, vitamin A-rich fruits and vegetables, other fruits and vegetables, and foods cooked with fat or oil	*√*	—	*√*	—	—	Y [31]

Diet Quality Index for Children; Kranz et al. 2004 [46]; 2–5 y Variety Index for toddlers	8: % total energy as added sugars, total fat, saturated fat, number of servings of grains, fruit and vegetables, dairy, excessive juice, and iron (mg/d) 5: bread group, vegetable group, fruit group, and dairy	—	—	—	*√*	*√*	N

(VIT); Cox et al. 1997 [47]; 2-3 y	Group, meat group	*√*	—	—	—	*√*	Y [20, 21, 26–28, 30]

Diet Quality Score 1 (DQS1), Canada; Campbell and Sanjur 1992 [48]; 2–4 y	6: milk, meat and alternatives, fruit and vegetables, breads and cereals, additional vegetables, and vitamin A-rich vegetables	*√*	—	*√*	—	*√*	N

Diversity Score (DS), USA; Caliendo et al. 1977 [36]; 2–4 y	1: dietary diversity using items consumed by 20% or more of the study samples. One point for every food item consumed from a list of 20 food items^b^	—	—	—	—	*√*	N

Table adapted from Smithers et al. 2011 [9].

Freq: frequency; FFQ: food frequency questionnaire; N: no; Y: yes.

Core foods: foods recommended to be consumed daily for example: fruit, vegetables, dairy, meat and alternatives, and cereals [6, 15].

Noncore foods: foods recommended to be consumed in minimal amounts for example: high fat, salt, and/or sugar foods [6, 15].

^a^If portion size-quantification required, index is only useful for data collected using semiquantitative or quantitative methods.

^b^Unlikely any short tool assess the same *x*-items.

^c^No tool assess bottle use.
