# Supplementary material for: Short Tools to Assess Young Children's Dietary Intake: A Systematic Review Focusing on Application to Dietary Index Research
Source: J Obes. 2013 Sep 26;2013:709626. doi: 10.1155/2013/709626 (PMC3807550; doi:10.1155/2013/709626)
Supplement: Supplementary file 1 — Supplementary 1 material provides details of the six-stage search process undertaken for the literature review. Details of inclusion and exclusion criteria applied to retrieved articles are detailed in the table. Supplementary 2 defines statistical tests used to assess reliability and validity of reviewed studies and highlights the review assessment criteria. [file 709626.f1.pdf]

Supplementary Information

Supplementary 1: Search process including search terms and exclusion criteria

Stage 1: Searching major science databases (search terms used)

1) Web of Science

Topic=((child* OR infant* OR toddler* OR preschool*))
AND Topic=((diet* OR food* OR nutrition OR dietary pattern OR eating pattern OR dietary intake OR food intake OR diet quality OR infant food OR infant nutrition OR child nutrition))
AND Topic=((assess* OR tool OR assessment tool OR dietary assessment OR questionnaire* OR evaluat* OR instrument OR checklist OR validit* OR correlat* OR compar* OR reproducibility OR accuracy))
Refined by: Topic=(assess) AND Research Areas=(NUTRITION & DIETETICS) AND Research Areas=(NUTRITION & DIETETICS OR PEDIATRICS) AND Research Areas=(NUTRITION & DIETETICS OR PEDIATRICS)
Timespan=All Years. Databases=SCI-EXPANDED, SSCI, A&HCI, CPCI-S, CPCI-SSH, CCR-EXPANDED, IC.

2) Pubmed

(((((((("child"[All Fields]) OR "infant"[All Fields]) OR "toddler"[All Fields]) OR "preschool"[All Fields])))
AND (((((((((("diet"[All Fields]) OR "nutrition"[All Fields]) OR "food"[All Fields]) OR "dietary pattern"[All Fields]) OR "eating pattern"[All Fields]) OR "dietary intake"[All Fields]) OR "food intake"[All Fields]) OR "diet quality"[All Fields]) OR "infant food"[All Fields]) OR infant nutrition) OR "child nutrition"[All Fields]))
AND ((((((((((("assess"[All Fields]) OR "tool"[All Fields]) OR "assessment tool"[All Fields]) OR "dietary assessment"[All Fields])) OR "checklist"[All Fields]) OR "reproducibility"[All Fields]) OR "valid"[All Fields]) OR "correlate"[All Fields]) OR "evaluate"[All Fields]) OR "food frequency questionnaire"[All Fields])
AND (Humans[Mesh]
AND English[lang]
AND (infant[MeSH] OR infant, newborn[MeSH] OR infant[MeSH:noexp] OR child, preschool[MeSH] OR child[MeSH:noexp]))))
AND ("assess"[All Fields])

3) Scopus

(TITLE-ABS-KEY((child* OR infant* OR toddler* OR preschool***))**
AND TITLE-ABS-KEY((diet* OR food***** OR nutrition OR dietary pattern OR eating pattern OR dietary intake OR food intake OR diet quality OR infant food OR infant nutrition OR child nutrition)) AND TITLE-ABS-KEY((assess* OR tool OR assessment tool OR dietary assessment OR questionnaire* OR evaluat* OR instrument OR checklist OR validit* OR correlat* OR compar* OR reproducibility OR accuracy)))
AND ORIG-LOAD-DATE AFT 1365250549
AND (LIMIT-TO(LANGUAGE, "English"))

Stage 2: Cleaning in Endnote

1) Duplicates were removed
2) Words were searched in TITLE and KEYWORDS to eliminate irrelevant articles

Abuse
Advert
Agriculture
AIDS
Alcohol
Anaemia / Anemia
Allergic/allergy
Anaphylaxis
Asthma
Atresia
Bacteria
burns
Case report
Case Study
Celiac disease / coeliac disease
Cerebral palsy
Colitis
Contamin (i.e. contamination)
Depress (i.e. depression)
Diabetes
Diarrhoea (diarrhea)
Dietary sup
Disabilit (I.e. disabilities, disability) / disabled
Disease
Disorder
Drug
Drug effects
Drug therapy
Down syndrome
Eating Disorders
Esophagitis
Epilepsy
Excretion
Exposure
Fertility
Fibrosis
Gastro
Genetic
Guideline
Heavy metal
HIV
Hormone
Hypertension
Illness
Immun (i.e. Immunology, Immunization, Immunisation)
In vitro
Infection
Injury
Intensive care
Intravenous
Leukemia
Malaria
Mental health
Meta-Analysis
Microb
Mice
Monkey
Otitis Media
Outbreak
Parenteral nutrition
Patient
Pesticide
Phenylketonuria
PKU
Pig
Poison
Practice Guidelines
Premature
Preterm infant / preterm
Primate
Rabbit
Rats
Renal
Retarded
Review
Rickets
Smoke
Soil
Steroid
Spina Bifida
Surgery
Syndrome
Therapy
Toxic
Transplant
Tube (i.e. neural tube defects, tube feeding)
Urinary
Urine
Vaccin (i.e. vaccinate, vaccine, vaccination)
Violen (i.e. Violence, Violent)
Virus

3) 'Cochrane' was searched in JOURNAL to eliminate review articles

Stage 3 and Stage 4: Inclusion and exclusion of articles

1) The resulting articles were screened firstly according to *title and abstract* (stage 3) and if uncertain, the *full text* (stage 4) was retrieved, using the screening process detailed in the tbale below.

Stage 5: Searching reference lists

1) Reference lists of all included articles and relevant reviews were searched for additional studies.

Stage 6: Updating search results

1) Stages 1-5 were re-run using articles published between 01.06.13 and 01.04.13 to identify any recently published short dietary assessment tools for 0-5 years that assess whole diet.

	EXCLUSION POINT						
	**1**	**2**	**3**	**4**	**5**	**6**	**7**
	**Study Outcomes**	**Study Assessment Method**	**Participants**	**Other**	**Dietary intake**	**Assessment of whole diet**	**Food items in short tool**
Included	Studies on individual dietary intake in infants and children (Outcome data includes nutrition-related measure - e.g. dietary intake, dietary patterns, energy intake, nutrient intake	Diet assessed using short dietary assessment tool<br>- Food Frequency Questionnaires<br>- other dietary questionnaires/ checklists	Healthy<br>- Infants and toddlers (1-23 months)<br>- Pre-schooler's (2-5 years)	- English language<br>- Humans	- Reported by primary caregiver/ parent	- Investigates intake of foods from all 5 food groups (fruit, vegetables, meat/alternatives, dairy, cereals) with/without high-fat/sugar 'extras' foods	- ≤50 items<br>-<br>*Items described as food intake questions. Excludes questions on dietary behaviour
Excluded	- Nutrition related outcomes such as nutrition knowledge, efficacy, attitudes, preference, behaviours (e.g. breastfeeding duration, weaning) etc.<br>- Group, school, family intake<br>Other studies, for example those<br>- Testing food/menu content e.g. additives, contaminants, nutritional content<br>- Describing supplement prescription and/or intake<br>- Measuring energy expenditure, urinary and blood concentrations of nutrients	Diet assessed using standard tool:<br>- dietary recall<br>- dietary record<br>- food diaries<br>- dietary indices<br>- dietary interviews<br>- large survey's	- Children and adolescents ≥5 years<br>- Diseased or institutionalis-ed subjects (e.g. Coeliac disease, CF, Cancer, FTT, Diabetes)	- Studies without an abstract<br>- NE = Language other than English<br>- NH = Not humans (animals)<br>- EO=excluded for other reason, describe here (e.g. review/report articles, study protocols)	- Reported by child	- Assesses intake of foods form <5 food groups e.g. only fruit and vegetable intake	- >50 items<br><br>*Items described as food intake questions. Excludes questions on dietary behaviours.
Code assigned	**NDI** = Not assessing dietary intake (no dietary intake outcome data)	**NST = Not Short Tool**	**NP = Not Population**	**NE** = Not in English<br>**NH** = Not humans<br>**NA** = No Abstract<br>**EO =** Excluded other	**NPR =** Not parent reported	**NWD =** Not whole diet	**NST50** = Not short tool ≤50 items

Supplementary Table: Definitions of review terminology and criteria used

Terminology	Definition	Criteria
Correlation coefficient (r)	A number, ranging from -1.0 to +1.0, used to describe the strength and direction of the linear relationship between two variables. Positive correlation; as one variable increases so too does the other. Negative correlation; as one variable increases the other decreases [1].	
Pearson's correlation coefficient	Correlation coefficient used for parametric data [1].	Low ≤0.50;<br>Moderate 0.51-0.69;<br>High ≥0.70
Spearman's correlation coefficient	Correlation coefficient used for non-parametric data [1].	Low ≤0.50;<br>Moderate 0.51-0.69;<br>High ≥0.70
Intra-Class correlation coefficient (ICC)	A measure of the reliability of measurements or ratings [2]. It is an explicit measure of the dependence of errors because it compares differences between groups to individual differences within groups [3]	Poor <0.50<br>Good ≥0.50<br>[4]
Kappa coefficient (k)	A measure of agreement between two different diagnostic tests for categorical data [1]. Kappa is 1.0 when agreement is perfect; it is 0.0 when agreement is no better than would be expected by chance [5].	Poor <0.20,<br>Fair 0.21-0.40<br>Moderate 0.41-0.60<br>Good 0.61-0.80<br>very good 0.81-1.00 [6]
Bland Altman analysis	Identifies the mean bias and 95% limits of agreement (± 2SD of the difference) between methods, providing an indication of whether a tool is valid for the assessment of intake at the individual and/or population level [7, 8].	-
Bland-Altman plot	A plot of the difference (bias) between two methods against the average of the two methods; used to evaluate strength of agreement [7, 8].	-
Sensitivity	Identifies the extent to which a diagnostic test correctly identifies those who have a particular condition or disease [9].	-
Specificity	Identifies the extent to which a diagnostic test correctly identifies those who *do not* have a particular condition or disease [9].	-
Cross-classification analysis	Identifies the percentage of subjects correctly or incorrectly classified by the tool into quartiles or tertiles of intake by the reference method.	

1. J. Pallant, "SPSS Survival Manual: A step by step guide to data analysis using SPSS". 4th ed. 2011: Allen & Unwin.
2. J. K. Peat, C. Mellis, and K. Williams, "Health Science Research: A handbook of quantitative methods". 2001, Victoria, Australia: Allen&Unwin.
3. B. Tabachnick and L. Fidell, "Using multivariate Statistics". 2007, USA: Pearson Education Inc.
4. A. M. Wilson, A. M. Magarey, and N. Mastersson, "Reliability and relative validity of a child nutrition questionnaire to simultaneously assess dietary patterns associated with positive energy balance and food behaviours, attitudes, knowledge and environments associated with healthy eating", *International Journal of Behavioral Nutrition and Physical Activity*, vol. 5, pp. 5, 2008.

5. W. P. Vogt, "Dictionary of Statistics & Methodology: A nontechnical guide for the social sciences". 2nd ed. 1999: SAGE Publications Inc.
6. D. G. Altman, "Practical statistics for medical research". 1991: Chapman & Hall/CRC.
7. J. M. Bland and D. G. Altman, "Comparing methods of measurement: why plotting difference against standard method is misleading", *Lancet*, vol. 21, no. 346(8982), pp. 1085-7, 1995.
8. J. M. Bland and D. G. Altman, "Measuring agreement in method comparison studies", *Statistical Methods in Medical Research*, vol. 8, no. 2, pp. 135-60, 1999.
9. P. M. Gleason, J. Harris, P. M. Sheean et al., "Publishing nutrition research: validity, reliability, and diagnostic test assessment in nutrition-related research", *Journal of the American Dietetic Association*, vol. 110, no. 3, pp. 409-19, 2010.
